# Efficacy of six disinfection methods against extended-spectrum beta-lactamase (ESBL) producing *E*. *coli* on eggshells *in vitro*

**DOI:** 10.1371/journal.pone.0238860

**Published:** 2020-09-11

**Authors:** Gerzon Motola, Hafez Mohamed Hafez, Sarah Brüggemann-Schwarze

**Affiliations:** Institute of Poultry Diseases, Department of Veterinary Medicine, Freie Universität Berlin, Berlin, Germany; Tokat Gaziosmanpasa University, TURKEY

## Abstract

The presence of extended-spectrum beta-lactamase (ESBL) producing *Escherichia coli* on poultry products is an important issue for veterinary and human health due to the zoonotic infection risk for producers and consumers. The present study focuses on testing the efficacy of six different disinfection methods on eggshell samples, aiming to reduce ESBL producing *E*. *coli* contamination on the hatching egg. Sterile eggshell cutouts were artificially contaminated with 10^8^ cfu/ml CTX-M-1 producing *E*. *coli* and used as a carrier model to analyze the efficacy of six disinfection methods. The contaminated samples were separated into two groups; 1) contaminated and disinfected, 2) contaminated and non-disinfected. Six independent disinfection protocols were performed following product specifications and protocols. Each eggshell sample was separately crushed, and the total viable bacterial count was calculated to determine the disinfection efficacy. Five out of six tested methods (formaldehyde gassing, hydrogen peroxide + alcohol spray, essential oils spray, peracetic acid foam, and low energetic electron radiation) demonstrated a reduction or completely eliminated the initial ESBL producing *E*. *coli* contamination. One method (essential oils as cold fog) only partly reached the expected efficacy threshold (reduction of >10^2^ cfu/ml) and the result differed significantly when compared to the reference method i.e. formaldehyde gassing.

## Introduction

Currently, poultry meat production (such as chicken, turkey, duck, and others) plays an important role in human food consumption, representing 12.7% of animal meat output in the EU [1].

The modern poultry industry is currently oriented to facilitate intensive meat production, producing high quality meat with a low economic cost. This, together with an increasing demand for poultry meat, eggs, and egg products, requires the constant maintenance of efficient hygiene regimes and goal-oriented animal healthcare to prevent the risk of contaminated food products. Vertical and horizontal infections with bacteria can be the cause of serious infectious poultry diseases which are frequently accompanied by heavy economic losses for the poultry industry [2].

The control and elimination of foodborne contaminants such as *Salmonella*, *Campylobacter*, and *E*. *coli* is an important challenge for producers [3, 4]. Bacterial contaminations occurring at any phase of the production chain can have severe implications on consumer health security. A recent report on extended-spectrum beta-lactamase (ESBL) producing *E*. *coli* on poultry products raised particular concern [5].

ESBL producing bacteria are Gram-negative bacteria that produce beta-lactamase, an enzyme that hydrolyses [6] the beta-lactam ring in antibiotics [7] such as penicillin and cephalosporin. This results in an acquired resistance against one or more third-generation beta-lactam antibiotics [8, 9]. The true prevalence of ESBL producing bacteria is not known [7], although studies refer to a high prevalence (up to 100%) of ESBL producing bacteria in animal productions in Germany [10–12]. The most frequent ESBL chromosomally encoded plasmids in human and veterinary medicine are SHV, TEM, and CTX-M [7, 13, 14]. The most common ESBL genotype present in poultry is CTX-M; which has a high affinity to cefotaxime [15, 16].

ESBL producing bacteria have been isolated in both human and animal hosts, and is also present in hospitals (patients and equipment), water, and on poultry products [17]. Not only human to human infections [13] but also zoonotic sources of ESBL producing bacteria [17–19] have been suggested. This underlines the importance to control their presence from the start of the poultry production chain: the hatching egg [20] Previous studies have demonstrated the transmission of ESBL producing *E*. *coli* along the broiler production chain, from grandparent flocks to the final meat product [21]. Interestingly, the prevalence of ESBL producing *E*. *coli* on hatching eggs is reported to be 1.8% of ESBL-/ AmpC beta-lactamases (pAmpC) -producing enterobacteria [22, 23], whereas 1-day-old broiler chicks have colonization rates up to 95% [24, 25]. Consequently, ESBL producing *E*. *coli* on the surface of hatching eggs are likely to be transferred to the hatchlings and eventually lead to a spread among animals at farm level [23, 24]. Furthermore, a pseudo-vertical transmission has been discussed, describing the transfer of bacteria from the hatchery environment to freshly hatched chicken [23].

Good hatching egg hygiene reduces the microbiota present on the eggshell surface, improves the chick quality [26], reduces mortality, and ensures an optimal production potential [27]. Formaldehyde fumigation has been used for more than 100 years as a method to remove bacteria from the surface of eggshells [28]. However, due to the potential carcinogenic, mutagenic, and toxic side effects of formaldehyde, the industry has been searching for alternative methods of disinfection [29, 30]. Alternative disinfectants containing hydrogen peroxide have demonstrated the potential to reduce bacterial contamination and improve hatchability [31, 32].

In this study, we tested six disinfection methods (conventional and alternatives) and their bactericidal efficacy against ESBL producing *E*. *coli*. Eggshell cutouts were used to investigate the disinfection efficacy on the matrix of interest, not only studying the superficial efficacy but also the disinfection effect on bacteria inside the pores [33, 34].

The efficacy of formaldehyde and five alternative disinfection methods was compared using a CTX-M-1 producing *E*. *coli* for the artificial eggshell contamination.

## Materials and methods

### Bacterial strain and growth conditions

For the artificial contamination, isolate No. 10682 (from one-day-old broilers) carrying a CTX-M-1 plasmid was kindly provided by the Institute for Animal Hygiene and Environmental Health, Freie Universität Berlin [10].

The isolate was inoculated in Luria-Bertani (LB) broth and incubated at 37°C for 24 hours. The culture was centrifuged at 5000 rpm for 15 min and the pellet was re-suspended in sterile phosphate-buffered saline (PBS) to achieve a suspension with 10^9^ colony-forming units per milliliter (cfu /ml). A 10^9^ cfu/ml suspension was required to reach a re-isolation rate of 10^5^−10^7^ cfu per eggshell from the eggshell carriers, as determined empirically during pre-trials.

### Eggshell carrier preparation

Non-incubated hatching eggs were obtained from a broiler breeder flock between the 14^th^ and 20^th^ week of production. Eggs were opened, the contents were decanted, and the shells were rinsed with flowing water. With a hand rotating tool (Dremel^®^) 2x2 cm squares were cut and the inner membrane removed. All eggshell carriers were placed in a glass Petri dish for sterilization using dry heat treatment at 180°C for 2 hours, and left to cool at room temperature in a sterile environment before use.

### Artificial contamination of eggshell carriers

Each trial comprised of three groups: A) non-contaminated, non-treated negative control, B) contaminated, non-treated positive control, and C) contaminated disinfected group. In each group, 10 eggshell cutouts were used as carriers, with the exception of low energy electron beam where only six eggshell cutouts were tested. The negative control (group A) was treated with sterile PBS (100μl per sample). Each carrier sample of groups B and C was artificially contaminated with 100μl of a 10^9^ cfu/ml bacterial suspension. The suspension was spread on the surface of the eggshell sample using a sterile inoculation loop. Samples of all groups were left to dry under a laminar airflow system (60 to 90 min). The aim was to achieve a contamination rate of 10^8^ cfu per eggshell to obtain a re-isolation rate between 10^5^–10^7^ cfu per eggshell in groups B and C.

### Disinfection of the eggshell samples

The contaminated and disinfected group (C) was treated with one disinfection method using the parameters and protocols provided from the producer (S1 Table). To avoid undesired mechanical wash off from the bacteria of the eggshell, disinfection was performed by avoiding spillage of the disinfection solution from the eggshell. Every method was tested three times with 10 samples in each trial. In the case of low energy electron beam, only two repetitions with six samples each were carried out, due to logistics limitations of the device management.

### Bacteria recovery and determination of bactericidal effect and disinfection efficacy

No stabilizer nor neutralization of the active disinfection substance was used for the bacterial re-isolation. Residue effects of products were not considered in this study. Each eggshell was crushed separately using sterile aluminium foil, collected in 1 ml sterile PBS, and mixed using a vortex agitator for 5 sec. A 10-fold serial dilution using PBS was performed for each sample and the drop plating method (10μl in triplicates) used for cfu determination on LB agar [35]. The agar plates were incubated at 37°C for 18–24 hours, and cfu were determined for each sample.

### Data analysis

For the efficacy analysis, each trial was evaluated separately, using the non-disinfected group (B) cfu mean as the base for the comparison of the re-isolation rate. The cfu value of every eggshell sample was compared with group B cfu mean. Available literature reports a prevalence of ESBL producing *E*. *coli* on the eggshell surface below 10^1^ cfu/ml [36]. In our studies (unpublished data), up to 10^2^ cfu/ml ESBL producing *E*. *coli* had been isolated from eggshell samples. Therefore, a reduction below the threshold of 10^2^ cfu/ml in the re-isolation was set as an expectation criterion for a successful disinfection to assure a complete disinfection of ESBL producing *E*. *coli* on eggshells. If more than 10^2^ cfu/ml were re-isolated from disinfected samples, the disinfection was considered incomplete and therefore unsuccessful. The efficacy of every method was calculated in percentage, representing the number of samples that reached the expected reduction of 10^2^ cfu/ml.

For the statistical analysis of the data normality of the continuous variables were investigated visually and using Shapiro-Wilk-test. Since data were not normally distributed, Mann-Whitney-U-test (2 groups) and Kruskal-Wallis-test (more than 2 groups) were used to investigate differences between groups. The statistical level of significance was 5 percent for all analyses (p ≤ 0.05). The effect size was calculated using the formula r = z/√N. R-value in the range 0.1<r<0.3 was considered as a low effect size, 0.3<r<0.5 as medium, and r>0.5 as a large effect size [37].

## Results

The re-isolation rate of the non-contaminated, non-treated negative control (group A) in all trials was 0 cfu/ml, proving eggshell cutouts were sterile, thus excluding unwanted contamination.

**Fig 1:**
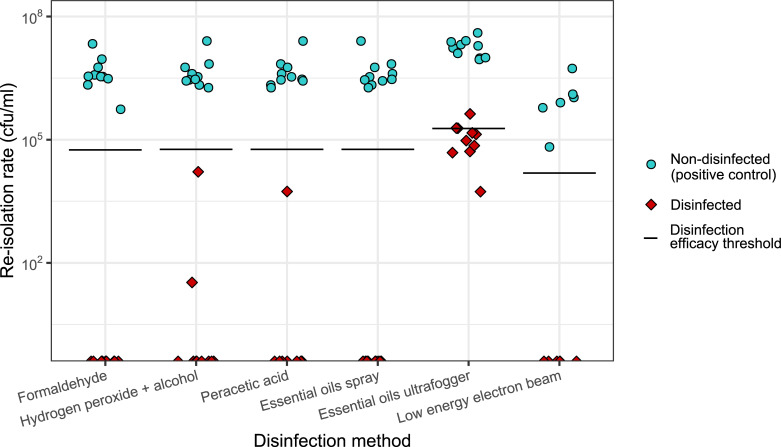
Bactericidal effect of six disinfection methods in an artificial ESBL *E*. *coli* contamination model. The bactericidal effect is presented in the reduction of the re-isolation rate of ESBL producing *E*. *coli* for each disinfection method. Each disinfection trial included 1) a non-disinfected positive control representing the initial contamination (blue data points), and 2) the disinfected group (red data points). Each point represents the value of one sample. For both groups n = 10, with the exception of low energy electron beam where both groups n = 6. The black horizontal line in each trial indicates the expected reduction of 10^2^ cfu/ml. Data representative for one of the three trials (see S1 and S2 Figs).

The re-isolation rate of the positive control (group B) in the six disinfection trials varied between 6.67 x 10^4^ cfu/ml and 4.00 x 10^7^ cfu/ml (Fig 1, blue data points). Comparing the re-isolation rates of the six disinfected groups (Fig 1, red data points), some methods achieved a complete elimination of the initial contamination, while others showed a reduction of the initial contamination with re-isolation rates up to 4.25 x 10^5^ cfu/ml (Fig 1, red data points). The re-isolation rates (in cfu/ml) of the six disinfection methods are presented in Fig 1.

Comparing the bactericidal effect of the six tested disinfection methods, we observed that formaldehyde, essential oils spray and low energy electron beam achieved a complete elimination of the artificial contamination. In the case of the essential oils spray, the surface of the eggshells after disinfection was oily, leaving oil residues after the crushing process.

In groups treated with hydrogen peroxide and peracetic acid, single outlier samples were detected with re-isolation values of 3.33 x 10^1^, 1.65 x 10^4^, and 5.48 x 10^3^ cfu/ml. These re-isolation rates still met the criterion of a successful disinfection (reduction of >10^2^ cfu/ml). Furthermore, the artificial contamination was eliminated in the majority of samples in the hydrogen peroxide and peracetic acid groups. Samples treated with essential oils ultrafogger demonstrated re-isolation rates in the range between 5.43 x 10^3^–4.25 x 10^5^ cfu/ml. These samples only partly met the criterion of a successful disinfection.

Taken together, all six disinfection methods showed significant differences when compared to the non-disinfected control group as well as a strong effect size (Table 1). However, comparing the differences in the bactericidal effect of the six disinfection methods among each other, they were not statistically relevant (Fig 1).

**Table 1:** Statistical data analysis of disinfected and non-disinfected groups for six disinfection methods.

Disinfection method	Group	N	Median	Range	Significance	Effect size
Formaldehyde	disinfected	10	0	0	<0.001^a^	0.902 ^b^
	non-disinfected	10	3.48x10^6^	2.12x10^7^		
Hydrogen peroxide + alcohol	disinfected	10	0	1.65x10^4^	<0.001^a^	0.873 ^b^
	non-disinfected	10	3.18x10^6^	2.35x10^7^		
Peracetic acid in micro-cages	disinfected	10	0	5.48x10^3^	<0.001^a^	0.886 ^b^
	non-disinfected	10	3.18x10^6^	2.35x10^7^		
Essential oils spray	disinfected	10	0	0	<0.001^a^	0.902 ^b^
	non-disinfected	10	3.18x10^6^	2.35x10^7^		
Essential oils ultrafogger	disinfected	10	1.15x10^5^	4.20x10^5^	<0.001^a^	0.845 ^b^
	non-disinfected	10	1.83x10^7^	3.10x10^7^		
Low energy electron beam	disinfected	6	0	0	0.002^a^	0.886 ^b^
	non-disinfected	6	9.35x10^5^	5.36x10^6^		

^a^ significant difference between groups

^b^ presents large effect size.

A p-value of <0.05 indicates statistical significance, and effect size interpreted as low (0.1<r<0.3), medium (0.3<r<0.5) and large (r>0.5).

In a second step, the efficacy of each method and trial was calculated. The efficacy of every method is presented as a percentage, representing the number of samples that reached the expected reduction of 10^2^ cfu/ml (Table 2).

**Table 2:** The efficacy of the tested disinfection methods presented as percentage from artificial contamination eggshell with ESBL producing *E*. *coli*.

	Disinfection efficacy in %		
Disinfection method / Trial	First trial	Second trial	Third trial
Formaldehyde	100%	100%	100%
Hydrogen peroxide + alcohol	100%	100%	90%
Peracetic acid in micro-cages	100%	100%	100%
Essential oils spray	100%	100%	100%
Essential oils ultrafogger	70%	40%	50%
Low energy electron beam	100%	100%	

Data of three independent trials (exception of low energy electron beam with only two trials).

The results show that formaldehyde, peracetic acid, essential oils spray and low energy electron beam had a 100% efficacy in all three trials. In the case of hydrogen peroxide + alcohol, there was a 90% efficacy in one trial and twice 100%, and essential oils with the use of an ultrafogger varied between 40% and 70%.

The capacity of each disinfection method to reduce the initial contamination was proven during the trials. All samples presented a reduction in bacterial re-isolation rates in comparison to the mean of the positive control.

The results of all tested methods were compared to the reference method of formaldehyde fumigation. The essential oils ultrafogger presented a significant difference (p-value <0.05), indicating that the group disinfected with Essential oils ultraffoger did not achieve a disinfection result comparable to formaldehyde. All the other disinfection methods presented no significant difference (p> 0.05) to the reference method formaldehyde.

## Discussion

A vertical transmission of bacteria into chicken eggs has been described as one of the crucial risks in egg and poultry production [38, 39]. Hatching eggs are usually incubated at 37°C in a high humidity environment. The egg contents provide plenty of nutrients for transmitted bacteria, representing optimal growth conditions for *E*. *coli* and other bacterial contaminants. To reduce transmission of bacteria (including ESBL producing bacteria), hatching eggs are routinely disinfected before incubation and during the hatching period.

Lately, reports on ESBL producing *E*. *coli* isolated from various poultry products [36, 40–42] have raised concerns regarding health risks for consumers [43–45]. To elucidate whether conventional disinfection methods and alternative methods are suitable for the elimination of ESBL producing *E*. *coli*, in this study, we tested six different disinfection methods *in vitro* for their efficacy against an artificial contamination with a CTX-M-1 producing *E*. *coli* strain. The use of eggshell samples served as an *in vitro* disinfection model. A relatively high contamination dose (10^6^−10^8^) was applied to the eggshell samples, and at the performance of the six methods investigated against the typical challenges when disinfecting eggs (e.g. shape, pores). Although previous studies report a presence of <10^1^ cfu/ml on eggshells of natural eggs [42], our studies (unpublished data) had registered a contamination up to 10^2^ cfu/ml of ESBL producing bacteria on the eggshell of hatching eggs. Therefore, a minimum reduction of 10^2^ cfu/ml was set as the expectation criterion for a successful disinfection. A reduction below 10^2^ cfu/ml was assumed to leave a residual contamination on the eggshell.

We tested conventional hatching egg disinfection methods in parallel with alternative methods: Formaldehyde fumigation is the standard method which is used in many hatcheries for hatching egg disinfection worldwide. The formaldehyde disinfection is efficient against a broad spectrum of bacteria [46, 47], hence making it suitable to treat hatching eggs with a diverse microbial community on the eggshell [48, 49]. According to our data, formaldehyde disinfection is highly effective (100% disinfection efficacy) against a relatively high (10^6^ cfu/ml) artificial ESBL producing *E*. *coli* contamination. Lately however, concerns have been raised regarding potential carcinogenic, mutagenic and toxic side effects for workers [50], with great speculation regarding future prohibition of its use [51]. Therefore, this study included five alternative disinfection techniques.

Hydrogen peroxide + alcohol has been used in hatcheries due to its easy application, and fewer hazardous characteristics [52], and its bactericidal effect against bacteria such as *Pseudomonas fluorescens*, *Proteus* sp. and *Staphylococcus aureus* [31]. The application requires a special nozzle that breaks the particles into smaller drops, giving the product the capacity to disinfect the eggshell surface and the pores. Previous studies have observed an effective disinfection against *Staphylococcus sp*. using this method [31]. During the eggshell trials presented here, two out of tree trials observed 100% disinfection efficacy (one trial with 90%). Consequently, this meant successful disinfection failed in 1/30 eggshell samples, taking all three trials into account.

Peracetic acid in micro-cages was successful in all three trials of this study, reaching a 100% efficacy against ESBL producing *E*. *coli*. Previous studies have indicated a positive disinfection effect of 1% peracetic acid solution against ESBL producing *E*. *coli* [53]. When working with this method, the strong, penetrative smell, and the potentially corrosive effect of the active substance also have to be considered. Under routine conditions, this application requires a well-ventilated environment and anti-corrosive surfaces. The foaming agent added to the product preparation used in this study allowed a prolonged exposure time and partly neutralized the strong smell of the peracetic acid. Past studies state un-efficacious disinfection against enterobacteria with peracetic acid [54] which might indicate that the foaming agent included in this study increased the disinfection efficacy against the chosen contamination model.

Essential oils are based on plant extracts and were selected as an alternative product to chemical disinfectants. Two different application methods were tested. The use of spray had a higher disinfection efficacy than the ultrafogger (cold mist) method. The spray method proved to be an easy application of the product, but it left a residual oil film on the samples, consequently leading to an obstruction of pores potentially interfering with the air exchange. In a follow-up study, this observation and its potential consequences for chick development and the hatching process were investigated. Indeed, a decrease in hatchability and a reduced body weight of hatched chicks was observed after the essential oil spray treatment [55]. Therefore, the essential oils spray treatment was deemed inappropriate in practice [55]. On the other side, the ultrafogger method did not leave any oil residue on the eggshell surface but did not provide a satisfactory disinfection rate, when compared to the reference method. The results obtained in this study were similar to published studies for other oil extracts [56]. Copur *et al*. reported that the use of oregano (Origanum onites L.) essential oil on the hatching egg surface negatively affected late embryonic development.

The low energy electron beam is a rather innovative approach that was tested in a prototype machine. To the authors’ knowledge, one publication has described the use of a similar technique to disinfect eggs until now [57]. Currently, no high throughput device for hatching egg disinfection is available. One limitation of the prototype was the small number of samples that could be decontaminated at a time. The results, however, were satisfactory showing 100% efficacy in both trials. This method clearly has a bactericidal effect against a relatively high (10^6^ cfu/ml) artificial ESBL producing *E*. *coli* contamination. The correct setting of the electron beam meeting the requirements of the sample is crucial when working with this technique [58]. For our study, we chose 60 kGy for the proper depth of the electron penetration and an intensity of 200 keV (S1 Table).

Taken together, our findings demonstrate that all six disinfection methods reduce the artificial ESBL producing *E*. *coli* contamination. This study adds important knowledge on the exact efficacy of conventional and alternative disinfection methods against ESBL producing *E*. *coli*. The statistical data analysis underlines no significant difference between the reference method formaldehyde and four alternative disinfection treatments (hydrogen peroxide + alcohol, peracetic acid in micro-cages, essential oils spray, and low energy electron beam). To be seriously considered as formaldehyde alternatives, the novel approaches need to prove practicability and suitability for high numbers of hatching eggs under field conditions. Further studies were performed in which potential side effects of the different disinfection methods on foetal development were evaluated, as well as effects on hatchability and the health status of one-day chicks [55].

## Supporting information

S1 Fig Second trial bactericidal effect of six disinfection methods in an artificial ESBL *E*. *coli* contamination model. The bactericidal effect is presented in the reduction of the re-isolation rate of ESBL producing *E*. *coli* for each disinfection method. Each disinfection trial included 1) a non-disinfected positive control representing the initial contamination (blue data points), and 2) the disinfected group (red data points). For both groups n = 10, with the exception of low energy electron beam where both groups n = 6. The black horizontal line in each trial indicates the expected reduction of 10^2^ cfu/ml.
(EPS)

S2 Fig Third trial bactericidal effect of six disinfection methods in an artificial ESBL *E*. *coli* contamination model. The bactericidal effect is presented in the reduction of the re-isolation rate of ESBL producing *E*. *coli* for each disinfection method. Each disinfection trial included 1) a non-disinfected positive control representing the initial contamination (blue data points), and 2) the disinfected group (red data points). For both groups n = 10. The black horizontal line in each trial indicates the expected reduction of 10^2^ cfu/ml.
(EPS)

S1 Table Disinfection methods used during trials. (DOCX)
