# Correction: Efficacy of six disinfection methods against extended-spectrum beta-lactamase (ESBL) producing *E*. *coli* on eggshells *in vitro*

**DOI:** 10.1371/journal.pone.0257120

**Published:** 2021-09-01

**Authors:** Gerzon Motola, Hafez Mohamed Hafez, Sarah Brüggemann-Schwarze

The following information is missing from the Funding statement: The publication of this article was funded by the OpenAccess Publication Fund of Freie Universität Berlin.
