# Supplementary material for: Efficacy of six disinfection methods against extended-spectrum beta-lactamase (ESBL) producing E. coli on eggshells in vitro
Source: PLoS One. 2020 Sep 11;15(9):e0238860. doi: 10.1371/journal.pone.0238860 (PMC7486133; doi:10.1371/journal.pone.0238860)
Supplement: S1 Table — (DOCX) [file pone.0238860.s003.docx]

**S1 Table. Disinfection methods used during trials.**

| Commercial name | Active substance | Application method | Concentration | Application protocol |
| --- | --- | --- | --- | --- |
| **Jäklechemie® Formaldehyd Biozid 20%** | Formaldehyde | Fumigation | 44 ml/m^3^ | 5 min fumigation + 10 min neutralization with ammoniac + 5 hours ventilation |
| **Wessoclean® K50 Goldline** | Hydrogen peroxide + alcohol | Fine Spray | Ready-to-use product | 1 min spraying + 1 hour exposure time |
| **Kesla ® 1+1 Wofasteril SC super** | Peracetic acid in micro-cages | Foam | 1% = 1 ml peracetic acid + 1 ml foaming agent | 1 hour exposure time |
| **Vitasan® Spray** | Essential oils | Spray | 5% mixed with distilled water | 20 min exposure time |
| **Vitasan® Spray** | Essential oils | Ultrafogger | 5% mixed with distilled water | 6 min fogging + 20 min exposure time |
| **Evonta® Ebeam Prototype** | Low energy electron beam | Radiation | 200 keV, 60 kGy | 1 sec exposure time |

Application protocols were according to the information provided by the producer.
